# Arrhythmic Mitral Valve Prolapse and Sports Activity: Pathophysiology, Risk Stratification, and Sports Eligibility Assessment

**DOI:** 10.3390/jcm13051350

**Published:** 2024-02-27

**Authors:** Paolo Compagnucci, Adelina Selimi, Laura Cipolletta, Giovanni Volpato, Alessio Gasperetti, Yari Valeri, Quintino Parisi, Antonio Curcio, Andrea Natale, Antonio Dello Russo, Michela Casella

**Affiliations:** 1 Cardiology and Arrhythmology Clinic, Marche University Hospital, 60126 Ancona, Italy; a.selimi@pm.univpm.it (A.S.); cipollettalaura@gmail.com (L.C.); giovol@live.it (G.V.); yarivaleri1@gmail.com (Y.V.); quintino.parisi@ospedaliriuniti.marche.it (Q.P.); antonio.dellorusso@gmail.com (A.D.R.); 2 Department of Biomedical Sciences and Public Health, Marche Polytechnic University, 60121 Ancona, Italy; agasper3@jhmi.edu; 3 Department of Cardiology, Johns Hopkins University, Baltimore, MD 21205, USA; 4 Division of Cardiology, Department of Pharmacy, Health and Nutritional Sciences, University of Calabria, 87036 Rende, Italy; 5 Texas Cardiac Arrhythmia Institute, St David’s Medical Center, Austin, TX 78705, USA; dr.natale@gmail.com; 6 Interventional Electrophysiology, Scripps Clinic, San Diego, CA 92037, USA; 7 Department of Internal Medicine, Metro Health Medical Center, Case Western Reserve University School of Medicine, Cleveland, OH 44195, USA; 8 Department of Medical, Special and Dental Sciences, Marche Polytechnic University, 60121 Ancona, Italy

**Keywords:** mitral valve prolapse, mitral regurgitation, arrhythmic mitral valve prolapse, sports cardiology, ventricular arrhythmias, risk stratification, electrophysiology study, electroanatomical mapping, physical exercise, guidelines

## Abstract

Although mitral valve prolapse (MVP) is the most prevalent valvular abnormality in Western countries and generally carries a good prognosis, a small subset of patients is exposed to a significant risk of malignant ventricular arrhythmias (VAs) and sudden cardiac death (SCD), the so-called arrhythmic MVP (AMVP) syndrome. Recent work has emphasized phenotypical risk features of severe AMVP and clarified its pathophysiology. However, the appropriate assessment and risk stratification of patients with suspected AMVP remains a clinical conundrum, with the possibility of both overestimating and underestimating the risk of malignant VAs, with the inappropriate use of advanced imaging and invasive electrophysiology study on one hand, and the catastrophic occurrence of SCD on the other. Furthermore, the sports eligibility assessment of athletes with AMVP remains ill defined, especially in the grey zone of intermediate arrhythmic risk. The definition, epidemiology, pathophysiology, risk stratification, and treatment of AMVP are covered in the present review. Considering recent guidelines and expert consensus statements, we propose a comprehensive pathway to facilitate appropriate counseling concerning the practice of competitive/leisure-time sports, envisioning shared decision making and the multidisciplinary “sports heart team” evaluation of borderline cases. Our final aim is to encourage an active lifestyle without compromising patients’ safety.

## 1. Introduction and Epidemiology

Mitral valve prolapse (MVP) is the most prevalent valvular abnormality in Western countries, affecting 2–4% of the general population [1,2,3]. MVP is defined as the systolic displacement of one or both mitral valve (MV) leaflets more than 2 mm above the mitral annular plane in the sagittal view of the MV at echocardiography [1]. The main etiologies are (1) myxomatous MVP or Barlow’s disease, (2) fibroelastic deficiency (FED), and (3) syndromic MVP in the context of connective tissue diseases such as Marfan syndrome, Loeys–Dietz syndrome, and Ehlers–Danlos syndrome [4,5]. Mitral annular disjunction (MAD) is a mitral annular abnormality that may not necessarily be associated with MVP and is described as a systolic separation or detachment of the basal left ventricular (LV) myocardium and the mitral annulus supporting the mitral leaflet (most commonly, the posterior leaflet), with a consequent abnormal systolic displacement of the leaflet hinge point into the left atrium (LA) [5,6].

MVP generally has a benign course [7], and subjects with MVP in the absence of moderate-to-severe/severe mitral regurgitation (MR) [8,9] or hemodynamic consequences on LV size and function reportedly have a similar life expectancy to the general population [10,11]. However, even in the initial description of MVP in the 1960s by JB Barlow and colleagues, it was recognized that a subset of patients with MVP have associated electrophysiological changes which may be recognized by 12-lead ECG, leading the authors to define an auscultatory-electrocardiographic syndrome, characterized by the association of mid-late systolic click, apical late systolic murmur, and abnormal Q waves and ST-T wave ECG changes, which were initially attributed to posteroinferior LV myocardial injury [12]. Fifty years of research in the field have confirmed that even among individuals with MVP and no/trivial MR or LV dysfunction, a small subgroup of patients may indeed be at high risk of malignant ventricular arrhythmias (VAs) and sudden cardiac death (SCD), and much attention has been devoted to the so-called arrhythmic MVP (AMVP) during recent years [13,14,15]. The classical AMVP phenotype is that of a female patient with bi-leaflet myxomatous degeneration and MVP, repolarization changes at 12-lead ECG, complex, polymorphic/right bundle branch block configuration VAs at Holter monitoring and exercise testing, and associated MAD with fibrosis of papillary muscles and posteroinferior LV [16].

The precise burden of SCD in MVP is unknown, and the yearly incidence of SCD among unselected patients with MVP (ranging from asymptomatic to severe MR and LV dysfunction) seems to be between 0.4% and 1.9% [13,14,15,16,17]. Conversely, the prevalence of MVP in SCD is 2% [15,17,18], but in autopsy studies, the prevalence of MVP among young patients with SCD rises from 4% to up to 12% [18,19,20], with MVP reported as the third most common cause of SCD (after arrhythmogenic right ventricular cardiomyopathy and coronary artery disease) in a clinical–pathological Italian registry of SCD < 35 years of age [21].

The prevalence of MVP among competitive athletes seems to be similar to that of the general population (2.9%), as reported by Caselli et al. [22]. In observational series of athletic cohorts, the incidence of SCD attributable to MVP ranged from 2% to 4% [23,24,25,26,27,28,29,30], similar to community-based studies of non-athletes. A recent meta-analysis on the causes of SCD in young athletes and non-athletes found that MVP was responsible for 3.6% of cases of SCD among athletes [31]. The main available studies concerning AMVP, SCD, and sports cardiology are noted in Table 1 [23,24,25,26,27,28,29], while the main clinical studies on clinical features of AMVP in athletic populations are noted in Table 2 [22,30].

Therefore, from a sports cardiology perspective, it is of paramount importance to comprehensively evaluate athletes with MVP to identify the high-risk AMVP subgroup and provide evidence-based advice concerning sports practice in order to prevent SCD. In this review, we summarize the current state of knowledge concerning AMVP, its relationship with sports practice, the clinical clues to identify patients at risk with non-invasive screening tests, and the role of additional evaluations for risk-stratifying patients, including electrophysiology study (EPS).

## 2. Definition and Classification of Arrhythmic Mitral Valve Prolapse

A recent European Heart Rhythm Association (EHRA) expert consensus statement on AMVP defines AMVP as the presence of MVP (with or without MAD) and frequent and/or complex VAs in the absence of other arrhythmic substrates, regardless of MR severity [5,32]. Complex VAs include ventricular fibrillation (VF), nonsustained, and sustained ventricular tachycardia (NSVT and VT), while frequent VAs mainly include premature ventricular complexes with a ≥5% burden at Holter monitoring [5,32]; at present, the authors of the expert consensus statement refrain from giving PVC morphology/origin-specific recommendations, to streamline the easy identification of AMVP patients in several clinical contexts and even outside referral centers. However, from a sports cardiology perspective, it appears of the utmost importance that in the presence of one or more PVCs at baseline 12-lead ECG or during exercise stress testing (which is mandatory in Italy for the assessment of competitive sports eligibility) several characteristics be assessed besides the presence and burden of PVCs [33,34]. These include QRS morphology (especially the presence of a non-fascicular [i.e., with duration ≥ 130 ms] right bundle branch block-like configuration), PVC polymorphism (at least two morphologies with each one representing ≥10% of PVC burden), precocity (the R-on-T phenomenon, couplets with short R-R interval), and PVC relationship with exercise (PVCs persisting or with increasing burden during maximal exercise stress testing), which should raise the suspicion of AMVP and mandate further assessments, including 24 h Holter monitoring with a training session, maximal ECG stress testing, and gadolinium-enhanced cardiac magnetic resonance imaging (CMR) [33,34].

Three main AMVP phenotypes have been recognized according to the main clinical context and pathophysiological mechanisms underpinning arrhythmogenesis [5]:

(1) AMVP due to moderate-to-severe/severe mitral regurgitation, in which hemodynamic mechanisms (especially LV volume overload) lead to increased risk of VAs and SCD [9], particularly in the presence of severe heart failure symptoms, reduced LV ejection fraction, and atrial arrhythmias. There is scientific evidence that relieving the hemodynamic burden by surgical mitral regurgitation correction may curtail the risk of both overall mortality and SCD, restoring a normal life expectancy [17].
(2) AMVP due to severe myxomatous MVP independent of MR severity or LV dysfunction. This phenotype often involves MAD, severe myxomatous degeneration with leaflet redundancy, excess leaflet length and thickness, and bileaflet MVP [5,32]. Importantly, the arrhythmic outcome of these patients is independent of gender, MR severity, LVEF, or bileaflet MVP [35] and seems to be associated with distinct substrates and triggers favoring arrhythmogenesis (see following paragraph);
(3) The combination of severe LA dilation (in excess of that expected for the degree of MR), atrial tachyarrhythmias (especially atrial fibrillation [AF]), and MVP may be defined as the atrial arrhythmia phenotype of AMVP. In the presence of severe LA dilation and AF, an excess mortality is commonly observed, independently of MR severity and LV dilation and/or dysfunction [36,37], and surgical MR correction may improve prognosis, highlighting the importance of recognizing this subset for informing management [10,11].

In the ensuing sections, we will focus on pathophysiology and risk stratification of the first two (i.e., the VA subset) clinical phenotypes from a sports–cardiology perspective, emphasizing the importance of clinical recognition with screening tests and the selected use of second- and third-level assessments.

## 3. Arrhythmogenesis in Mitral Valve Prolapse and Relationship with Physical Exercise

**Figure 1:**
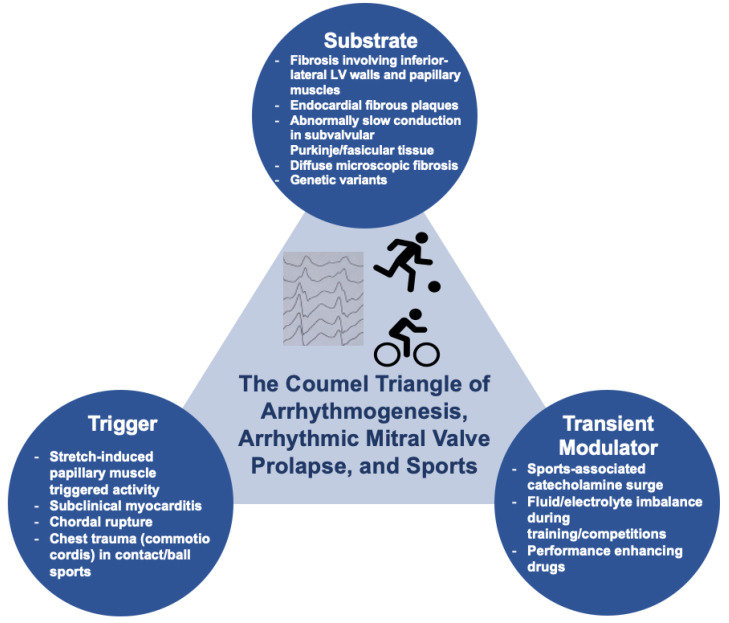
Pathophysiology of AMVP and its complex interplay with sports activity.

Several mechanisms may play a role in the genesis of VAs in the context of MVP, and these mechanisms may be facilitated by physical exercise. However, the relative contribution of individual factors may be difficult to ascertain in individual cases. By analogy with the Coumel triangle of arrhythmogenesis, Miller and colleagues proposed that VAs in MVP are the result of the complex interplay among three factors: a substrate, a trigger, and a transient modulator [38]. We will examine individual factors in the following sections (Figure 1).

### 3.1. Abnormal Myocardial Substrate

In a 2015 autoptic case series including 43 SCD victims in whom MVP represented the only cardiac abnormality identified at autopsy, Basso et al. provided the histological evidence of endoperimysial and/or patchy replacement-type myocardial fibrosis involving the papillary muscles (100% of the study cohort), adjacent LV free wall, and infero-basal LV free wall (88% of the study cohort) under the posterior MV leaflet, with subendocardial–midmural layer distribution [19]. Fibrosis in these particular regions was hypothesized to be the result of abnormal tension and excessive mechanical stretch/movement of papillary muscles and infero-basal LV myocardium caused by prolapsing leaflets, elongated chordae [16,19,32], and MAD [35,38,39], which may, in turn, be the result of primary abnormalities of these valvular and surrounding tissues. These abnormalities tend to progress due to the repeated mechanical load on the MV apparatus, which is exposed to the systolic arterial pressures developed by the LV from beat to beat, favoring a vicious cycle of mechanically induced myocardial damage, replacement-type fibrosis, and valvular apparatus dysfunction aggravating the mechanical load on the perivalvular myocardium. In a seminal review article on MVP and SCD, Basso and colleagues proposed that MAD may be regarded as the primary initiating factor for the cascade of events leading to myocardial fibrosis and arrhythmogenesis in AMVP, the so-called Padua hypothesis [16]. Although intriguing, further research should be conducted to definitively validate it. This pathophysiological process may be compounded by endocardial fibrous plaques, which are supposed to be the result of the friction between the elongated and stretched chordae tendinae and the posterolateral LV endocardium and which were reported in 58% of the cohort described by Basso et al. [19]. In the same work, the histological finding of papillary muscle and infero-basal fibrosis was confirmed in vivo using CMR in 93% of a cohort of AMVP patients (*n* = 30) versus a 13% prevalence in a control group of MVP without complex VAs (*n* = 14), highlighting the link between the abnormal fibrous LV substrate and arrhythmogenesis [19]. The pathophysiological relevance of LV replacement fibrosis and its strong association with the AMVP phenotype has been reinforced by two subsequent studies.

In the former, Kitkungvan and coworkers described a cohort of patients with primary MR (*n* = 156), with or without MVP, who underwent CMR. They found that MVP was the strongest predictor of CMR-proven replacement-type fibrosis, even after adjustment for clinical data and the severity of MR. Interestingly, MVP patients had a peculiar regional distribution of fibrosis in the inferolateral LV wall; the prevalence of fibrosis was markedly higher at any degree of MR severity in MVP compared to non-MVP patients, and MVP patients with trace MR had a higher prevalence of fibrosis than non-MVP patients with severe MR. Myocardial fibrosis was, in turn, the variable with the strongest association with major arrhythmic events in univariable analysis, with the highest rate of events seen in patients with both MVP and CMR-proven fibrosis (7.7%), followed by patients with MVP without fibrosis (2.7%), and non-MVP patients (0.6%, *p* < 0.01) [40].

In the latter work, Constant Dit Beaufils and coworkers conducted a dual-center, observational, prospective study in which 400 patients with MVP undergoing comprehensive echocardiography and CMR were included. The authors described an incremental association between the degree of MR and the prevalence of CMR-proven myocardial fibrosis, which mainly involved the basal inferolateral segment with midmural distribution and papillary muscles. Furthermore, patients displaying replacement-type fibrosis had more dilated LV, greater LV mass, more remodeled MV apparatus, and higher prevalence of VAs, supporting the concept that the chronic volume overload induced by hemodynamically significant MR may aggravate myocardial damage and fibrosis in MVP. However, the authors also separately analyzed the subgroup of MVP patients with trace/mild MR and found that 16% of these patients had abnormal LV dilation (which was disproportionate to the degree of MR) and that 13% had replacement fibrosis. In the trace/mild MR subset, fibrosis was associated with more abnormal features of the MV apparatus and a higher prevalence of VAs. In the whole cohort, CMR-proven fibrosis was found to be independently associated with a higher risk of a composite arrhythmic/non-arrhythmic endpoint during follow-up [41].

These two studies in combination seem to support the hypothesis of an “MVP-associated cardiomyopathy”, according to which the stretch- and elongation-induced fibrosis of the inferolateral LV wall and papillary muscles are strongly associated with the MVP phenotype (and even more with AMVP), it may be found early in the clinical course of MVP and tends to be further aggravated by the hemodynamic progression of MR [40,41]. The role of genetic and inflammatory mechanisms in the determinism and progression of MVP-associated cardiomyopathy and of the AMVP phenotypes are still to be elucidated but may be relevant according to case series and small reports describing familiar clustering and fluctuating inflammation-dependent clinical course [42,43]. As far as genetics are concerned, variants in the FLNC, LMNA, and HCN4 genes have been described in small case series of familiar AMVP [42]. Interestingly, FLNC and LMNA are implicated in the pathogenesis of cardiomyopathies characterized by extensive myocardial replacement-type fibrosis and VAs; however, the causal (i.e., signal of a possible pathophysiological link) versus casual (i.e., noise due to the high prevalence of MVP in the general population) association of these genetic variants is still to be confirmed, and further studies are highly needed to elucidate the role of genetic variants in the development of an AMVP myocardial substrate [42]. Even for inflammation, supporting evidence is limited to isolated case reports [43]. However, inflammation may render the ventricular myocardium more vulnerable to VAs [44], and this pathophysiological pathway may be especially relevant in elite athletes, who are at risk of viral infections due to frequent international travels, outdoor training competitions, exposure to extreme climates, and exercising despite symptoms of common cold, which may all facilitate the development of myocarditis [45,46], especially in the COVID-19 era [47].

### 3.2. Trigger

The mechanical stretch of papillary muscles may also represent the trigger for malignant VAs by causing electrophysiological changes such as shortening of the action potential duration, decrease in the resting diastolic potential, prolongation of ventricular functional refractory period, and development of early afterdepolarizations resulting in triggered activity [48,49,50]. It is therefore not surprising that in AMVP, VAs commonly originate from papillary muscles, which are enriched in Purkinje fibers capable of automaticity and triggered activity [19,51]. Interestingly, in a study including six patients with MVP and prior cardiac arrest, Syed et al. demonstrated that PVCs preceded by Purkinje signals (mapped to the papillary muscles in four cases) acted as VF triggers [52]; furthermore, all these patients had evidence of abnormal Purkinje electrograms (fractionated, split, and delayed potentials) suggesting abnormal Purkinje tissue conduction [52]. In addition, the presence of Purkinje potential at the ablation site was associated with procedural success [52]. Although during EPS, other sites of origin beyond papillary muscles have been recognized, including mitral annulus, fascicles, outflow tracts, and basal septum, all these regions are enriched in subendocardial Purkinje network, supporting the concept of microstructural damage to the Purkinje tissue underlying PVCs in AMVP [52,53]. An otherwise innocent PVC may initiate sustained reentry degenerating in VF in the presence of myocardial fibrosis, and this phenomenon may occur more commonly when the PVC is short-coupled (i.e., R-on-T phenomenon) [54]. It is unlikely that the occurrence of Purkinje tissue damage/ectopy in association with MVP and myocardial fibrosis could be casual [52]; the subendocardial Purkinje network at LV subvalvular sites may develop microstructural damage as a consequence of stretch/elongation-induced mechanical damage, which also leads to myocardial fibrosis; in alternative, Purkinje tissue damage may be a result of a primary tissue abnormality related to the AMVP phenotype [52]. Whether the practice of high-intensity sports may aggravate Purkinje tissue damage and facilitate ectopies remains an unanswered question, which would require a prospective assessment of the effects of sports in long-term follow-up studies of MVP patients (see the paragraph on exercise prescription and sports eligibility assessment).

### 3.3. Transient Modulator

Finally, transient increases in sympathetic tone may represent the critical factor precipitating SCD in AMVP, especially during the practice of sports [38,55]. Previous work has demonstrated an association between MVP, reduced vagal tone, and increased sympathetic activity [38]; furthermore, papillary muscle stretch may lead to spontaneous depolarization of myocardial nerve endings via mechanoelectrical feedback [56], leading to changes in the central nervous system activity and further release of catecholamines, which may in turn increase calcium release from the myocyte sarcoplasmic reticulum, modulate membrane ion channels, and favor afterdepolarizations, especially in the presence of genetic polymorphisms of the β1 adrenergic receptor leading to hypersensitivity to catecholamines [57]. These autonomic mechanisms may be especially relevant for the risk of SCD associated with sports activity in AMVP patients [58]. Finally, the use of performance-enhancing drugs/substances, which is especially common among recreational athletes, may represent another sports-specific transient factor favoring arrhythmogenesis and SCD due to PVC-initiated VF in the presence of perivalvular myocardial fibrosis and Purkinje tissue disease, although this hypothesis lacks validation in properly conducted clinical studies [59].

## 4. Phenotypic Characterization and Risk Stratification

The EHRA consensus statement on AMVP recommends a structured risk stratification process aimed at identifying AMVP and assessing the risk of SCD [5]. A similar approach should be applied to the assessment of the athlete with MVP, as recommended in both international and Italian guidelines on sports eligibility [58,60,61].

**Figure 2:**
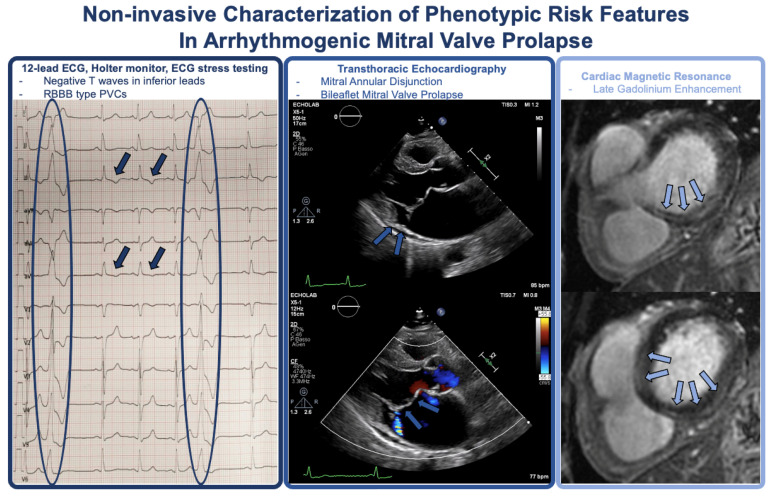
Phenotypic features of AMVP at non-invasive tests.

A comprehensive risk stratification of patients with MVP is mandatory and should start with clinical history, ECG, and exercise stress testing (especially in Italy, where exercise stress testing is mandatory for the assessment of competitive sports eligibility) [61]. In the presence of suspicion for AMVP, 24 h Holter monitoring, maximal ECG stress testing (if not previously performed), and echocardiography should be used to identify factors associated with increased risk of VAs and SCD [5,61]. Cardiac magnetic resonance, continuous rhythm monitoring with implantable loop recorders (ILR), and electrophysiology study may be performed to further refine risk stratification in selected cases (Figure 2) [5,60,61].

At the same time, the use of further diagnostic tests in asymptomatic patients without other features suggesting increased arrhythmic risk (which may represent the majority of the MVP population) may not be appropriate and lead to increased costs, inappropriate referrals, and false positive results potentially limiting participation in sports, with psychological, health, and economical consequences [60,61,62].

### 4.1. History and Physical Examination

Patient assessment should start with the assessment of family history of MVP and/or SCD/major VAs. The propensity for familiar association of MVP should dictate the echocardiographic evaluation of first-degree relatives of patients with MVP and severe MR undergoing surgery [63].

A second fundamental step is the ascertainment of symptoms, including syncope, pre-syncope, and palpitations. The risk of VAs and SCD is not uniform among patients with AMVP but ranges from very high risk in patients recovered from sudden cardiac arrest, high risk in patients with unexplained syncope, and low risk in asymptomatic patients [5,64]. Syncope was reported in 35% of MVP patients with malignant arrhythmias or SCD [65]; thus, particularly if unexplained or occurring during exercise, in the sitting/supine position, without prodromal symptoms, or when preceded by palpitations should raise the suspicion for malignant VAs and dictate a more intense screening with 24 h Holter monitoring and exercise stress testing at a minimum [5,60,61], and mandate caution for the assessment of sports eligibility. A similar approach may be considered for patients with pre-syncope. Patients with MVP frequently experience palpitations and chest pain; although these symptoms do not seem to have discriminative value in identifying patients at high risk of SCD per se [35], they need to be carefully assessed. Twenty-four-hour Holter monitoring and maximal ECG stress testing should be performed in athletes with known or suspected MVP in the presence of palpitations, even when VAs are not evident at 12-lead ECG [61].

The physical examination retains paramount importance in the setting of preparticipation screening. The association of mid-late systolic click and late systolic murmur with typical variation in response to changes in preload/afterload may be the only clue to MVP, and auscultation/pulse palpation may reveal otherwise unrecognized premature beats [62]. Patients should be evaluated for possible phenotypic features of Marfan syndrome (tall stature, pectus carinatum, arm span larger than height, arachnodactyly of hands and feet), according to the revised Ghent criteria, which can be used to diagnose the syndrome [62].

### 4.2. Twelve-Lead ECG and Holter Monitoring

The 12-lead ECG may reveal typical abnormalities, which may suggest an AMVP phenotype and need for further diagnostic evaluations.

T wave inversion or biphasic T waves, often in inferior and lateral leads, are independently associated with the AMVP phenotype and malignant VAs/aborted SCD [18,19,66,67]. These abnormalities seem to be related to fibrosis and altered contractility/stretch of papillary muscles and LV infero-basal segment, which may result in repolarization abnormalities, as described in the previous section [5]. In a recent study evaluating the electrophysiological substrate in MVP patients, low unipolar voltage areas (<8.3 mV) in the sub-mitral region of the LV inferior or lateral wall and papillary had a greater extension in patients with negative T waves [68]. Other risk features may be recognized and assessed at 12-lead ECG, including QT interval and QRS fragmentation, but their prognostic importance in AMVP remain speculative [5].

Additionally, 12-lead ECG may also reveal VAs, which are assumed to have a high burden in case they can be captured in repeated ECGs [5]. Besides NSVT/VT, which can be rarely documented in the absence of protracted monitoring but may indicate higher risk, PVCs with right bundle branch block configuration may typically arise from papillary muscles or other peri-mitral structures [19,51,52,53] and require further evaluations with 24 h Holter monitoring, maximal ECG stress testing, echocardiography, and also CMR in athletes seeking advice regarding sports participation [61]. Due to the importance of identifying the AMVP subgroup, authors of the EHRA expert consensus statement suggested that all patients with MVP undergo Holter monitoring, although the cost-effectiveness of such a recommendation has not been well evaluated in the context of a very common (and most often benign) condition such as MVP [5].

Although PVCs originating from the PMs/fascicular system and from Purkinje tissue may act as potential triggers for VF [52,54,69], knowledge about the prognostic significance of the various morphologies/origins in AMVP is currently limited and warrants further research [5]. Regarding the importance of VA complexity, Essayegh et al. found that the severity of arrhythmia (VT ≥1/min) was independently associated with a higher risk of death after adjustment for baseline features and MVP characteristics in a large cohort of MVP patients undergoing comprehensive evaluation [35]. Consequently, authors of the EHRA expert consensus statement proposed to classify VAs into different risk categories [5]:

(1) High-risk VAs: sustained VT not originating from the right or LV outflow tract, spontaneous polymorphic NSVT, and rapid monomorphic NSVT (>180 bpm);
(2) Intermediate-risk VAs: polymorphic PVCs, monomorphic NSVT at a lower rate (<180 bpm), and highly frequent or complex PVCs (bigeminism and couplets);
(3) Low-risk VAs: frequent PVCs (≥5% total PVC burden) but not complex VAs (and no morphological features suggesting higher risk categories—i.e., patients with monomorphic outflow tract PVCs).

While it was proposed that patients with high-risk VAs may be considered candidates for implantable cardioverter defibrillator implantation [5], and it is advisable that patients with both high- and intermediate-risk VAs refrain from practicing high-intensity sports; patients in the low-risk category may benefit from regular reassessment and continued sports practice [61].

Periodic 24 h Holter monitoring of arrhythmia burden and complexity is useful for risk reclassification given the possibility of disease progression, and although the optimal frequency of monitoring is not well established, it should be tailored to the risk category, with 6-month/yearly evaluation in intermediate-risk patients and every 2–3 years in low-risk subjects [5]. Longer ECG monitoring (up to 7 days) may have a role in case patients’ symptoms (pre-syncope or palpitations) remain unexplained after 24 h ECG monitoring.

According to the EHRA expert consensus, the implantable loop recorder (ILR) can be useful in several clinical contexts [5]:

- In patients with unexplained syncope or pre-syncope without high-risk VAs at Holter monitoring;
- In cases of high-risk features, negative CMR, and without indications for an ICD (hemodynamically tolerated VT, NSVT);
- In patients with phenotypical risk features (T-wave inversion in the inferior leads, repetitive documented polymorphic PVCs, MAD, redundant MV leaflets, enlarged left atrium or ejection fraction ≤ 50%) plus positive LGE on CMR.

These recommendations are mainly based on a recent prospective follow-up study of 80 patients with AMVP undergoing continuous rhythm monitoring using ILR and ICD, which found that the yearly incidence rate of first severe VA in the ILR group was 4%, while it was 8% for re-events in the ICD group. Frequent PVCs, NSVTs during follow-up, greater LV diameter, and longer posterolateral MAD distance predicted the first severe VA in the ILR group [70].

### 4.3. Signal Averaged Electrocardiogram

Late potentials on signal-averaged ECG (SAECG) are low-amplitude, high-frequency signals that reflect slow and fragmented myocardial conduction related to arrhythmogenic substrates. SAECG abnormalities are associated with an increased risk of ventricular arrhythmias and sudden cardiac death in both ischemic and non-ischemic cardiomyopathies, arrhythmogenic right ventricular cardiomyopathy, and Brugada syndrome [71]. Studies involving SAECG showed an increased frequency of late potentials in MVP patients but without clear risk prediction [72,73,74,75,76,77]. These studies concluded that late potentials are a common finding in patients with MVP and that they may be regarded as a risk modifier in conjunction with other clinical/imaging findings [15,72,73,74,75]. To the best of our knowledge, the are no studies comparing late potentials on SAECG and areas of scar/late potentials at EAM in AMVP patients; however, as previously proposed in the setting of ischemic and nonischemic cardiomyopathy [78,79], we may speculate that late potentials at SAECG may be included among the risk markers (i.e., syncope or family history of SCD) that, if present, may suggest the application of a two-step approach in risk stratification, using EPS in patients displaying a higher risk profile.

### 4.4. Exercise Stress Testing

Exercise stress testing is an important tool to assess suspected exercise-induced VAs [80], as several reports have established exercise-induced VAs as a predictor of cardiovascular mortality even in asymptomatic patients [81,82,83], although there are no data on the significance of these arrhythmias specifically in patients with AMVP. The EHRA expert consensus statement on AMVP suggests exercise stress testing in order to assess adrenergic-dependent arrhythmias and exercise tolerance in AMVP patients [5]. From a sports cardiology perspective, exercise-induced VAs or VAs persisting during maximal exercise stress testing may suggest higher sports-related arrhythmic risk and warrant 24 h Holter monitoring, echocardiography, and CMR to rule out other risk features, especially when VAs are reproducible in two distinct tests [33,34,35,36,37,38,39,40,41,42,43,44,45,46,47,48,49,50,51,52,53,54,55,56,57,58,59,60,61]. Consistently, a recent study on the clinical value of the preparticipation screening protocol in Italy (which includes personal/family history taking, physical examination, resting 12-lead ECG, and exercise stress testing) showed that among the three patients diagnosed with AMVP, there were abnormal findings at exercise stress testing in each case (100% sensitivity), while resting ECG was always normal (0% sensitivity), and physical examination was unremarkable in one subject (66% sensitivity), potentially supporting the importance of exercise testing in unmasking the AMVP phenotype [84].

### 4.5. Transthoracic Echocardiography (TTE)

In patients with suspected AMVP, a comprehensive TTE assessment following guideline-based imaging protocol is required, with qualitative, semiquantitative, and quantitative evaluation of MR for severity grading [10,85]. TTE allows for the differentiation of fibroelastic deficiency and Barlow’s disease by morphologic characteristics of the MV apparatus. In FED, echocardiography shows normal or thin leaflets without redundant tissue, usually segmental prolapse, chordal rupture, and normal or mild annular dilatation, whereas in Barlow’s disease, it shows thickened and redundant leaflets with bileaflet prolapse or prolapse of multiple segments, chordal elongation rather than rupture, and severe annular dilation [86]. MAD presence should be noted, and MAD length should be measured in the parasternal long-axis view in systole, although there are no clear cut-offs of severity [5]. MAD is associated with LV hypertrophy and fibrosis as a consequence of excessive leaflets’ mobility caused by systolic curling and annular detachment [39] and with LV enlargement in excess of MR [87], as previously discussed.

**Figure 3:**
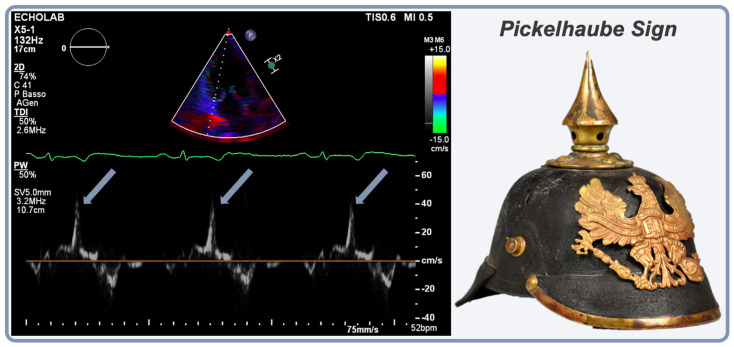
Pickelhaube sign, a distinctive spike during systole of the lateral mitral annulus using tissue Doppler.

Studies investigating AMVP found that the most frequent echocardiographic morphologic features of the typical AMVP are severe myxomatous degeneration with bileaflet MVP (Barlow’s disease) and MAD [16,19,35,39,66,87]. Although highly prevalent, a large cohort study showed that bileaflet MVP is not independently an SCD trigger [8], whereas another cohort study showed that MAD with MVP is independently associated with ventricular arrhythmias at diagnosis or developing during follow-up [87], independently of MR severity [35]. A recent meta-analysis confirmed that MVP patients with MAD had a higher risk of VA, with a risk ratio of 1.90 [88]. Furthermore, a distinctive spike during systole of the lateral mitral annulus using tissue Doppler, called the Pickelhaube sign, has been associated with MAD (Figure 3) [89]. The mechanism hypothesized is related to the movement of the prolapsing leaflet in mid-systole that tugs the posteromedial PM, thus pulling the adjacent posterobasal LV wall toward the apex, resulting in the spiked configuration of the lateral annular velocities [89]. The Pickelhaube sign has been regarded as a marker of a more severe AMVP phenotype since its initial description, being associated with a higher prevalence of VF, abnormal 12-lead ECG, and CMR-proven fibrosis, and should therefore be searched for at the time of echocardiographic assessment [89].

Besides MV apparatus morphology and function, left-sided chamber dimensions and function and LA volume should be carefully assessed [62]. An LV ejection fraction <60% should be considered abnormal in the presence of moderate-to-severe or severe MR. LV dilation should be noted and discriminated from the harmonic (i.e., involving all cardiac chambers homogeneously) chamber dilation seen in athletes’ hearts among endurance athletes. In the presence of moderate-to-severe or severe MR, LV end-diastolic diameter (LVEDD) > 60 mm (or >35.3 mm/m^2^ in men and 40 mm/m^2^ in women) should be considered abnormal, especially if associated with LVEF < 60% and pulmonary artery hypertension [60,62].

Even for TTE, due to the possibility of structural progression, the examination should be repeated at regular intervals, at least yearly, for competitive athletes [61,62].

### 4.6. Cardiac Magnetic Resonance

Cardiac MR with LGE sequences is an important tool for arrhythmic risk stratification of VAs in patients with MVP, allowing the detection of focal myocardial fibrosis, which is present in 28–37% of patients with MVP [40,41]. Several studies have shown that fibrotic changes in MV apparatus, including LGE at the mid-wall of the PMs and patchy LGE in the LV infero-basal myocardium, have a clear pathophysiological association with complex VAs, as previously discussed [19,39,40,41,90]. The assessment of diffuse interstitial fibrosis using T1 mapping (significantly shorter post-contrast T1 time) has suggested an association with increased risk of complex VAs even in the absence of focal fibrosis [91]. Additionally, CMR provides a detailed assessment of MV anatomy and can detect MAD and assess its severity and extent [5,92]. According to a CMR study, although MAD occupied almost half of MV circumference (median value, 150°), with common involvement of anterior and inferior walls, only posterolateral MAD distance and papillary muscle fibrosis were significantly associated with a severe arrhythmic presentation at multivariable analysis [92].

According to the EHRA expert consensus on AMVP, CMR should be performed in all the following clinical contexts [5]:

- After aborted SCD or sustained VAs, before implanting an ICD;
- In patients with a history of unexplained syncope or documented NSVT;
- In the case of AMVP and at least one phenotypical risk feature;
- In patients in whom echocardiography does not provide an accurate assessment of left/right ventricular function or of MV characteristics.

Similarly, according to Italian guidelines for the assessment of sports eligibility, CMR should cautiously be performed in the presence of phenotypic risk features (family history of SCD, moderate or severe MR, MAD, bileaflet MVP, syncope/pre-syncope, complex PVCs, NSVT/VT, and LV dilation or dysfunction), in order to identify or rule out myocardial fibrosis, which may further refine the sports eligibility assessment [61]. However, for a rational use of resources, the clinical evaluation of the athlete with known or suspected MVP should always start with first-line tests (physical examination; 12-lead ECG), includes a comprehensive echocardiographic assessment, and a maximal ECG stress testing, with no need to proceed to CMR in the absence of phenotypical features associated with AMVP [33,61].

### 4.7. Electrophysiological Study and Electroanatomical Mapping

The role of electrophysiology study (EPS) in risk stratification of patients with AMVP is controversial, and the EHRA expert consensus statement on AMVP does not endorse the use of EPS with programmed ventricular stimulation (PVS) for risk stratification [5]. In fact, a systematic review by Han et al. reported on the outcome of EPS with PVS in 22 patients with MVP and prior aborted SCD: in only 1 of the 22 cases (5%), a sustained monomorphic VT could be induced, suggesting that arrhythmia mechanism is PVC-triggered rather than reentrant, potentially making PVS less useful for risk stratification in this setting [65].

A recent study by Vergara et al. showed that EPS sensitivity for SCD was low, with only one patient with prior aborted SCD (25%) presenting positive PVS; therefore, the authors concluded that the usefulness of PVS alone for risk stratification in patients with AMVP seems to be limited [68]. Another study by Marano et al. on long-term outcomes of ablation for VAs showed that all the patients (5/5) that had sustained VA during 9 year follow-up after the index ablation had evidence of sustained VT inducibility during index EPS with PVS performed prior to ablation [53]. Since all these patients had complex multifocal PVCs, induction of sustained VT may represent, together with multifocal PVCs, a useful indicator of risk for progressive arrhythmias, for which earlier ICD implantation may be beneficial [53].

Miller et al. [38] suggested performing EPS for risk stratification in patients with multiple phenotypic risk factors, such as the evidence of a trigger (pleomorphic PVCs) and substrate (CMR-proven myocardial fibrosis) for SCD. If carried out, a standard PVS protocol should be used, including up to three extra-stimuli from two different sites (right ventricular apex and right ventricular outflow tract) down to effective refractory period (ERP) or 200 ms [5]; the use of isoproterenol to increase the diagnostic yield of the test is currently not supported by the available literature [93]. The induction of polymorphic VT and VF with aggressive stimulation protocols (with three extra-stimuli) is a nonspecific finding and may not predict the future risk of SCD [38]. In patients with a “positive” EPS (sustained monomorphic VT induction), Miller et al. recommended implantation of an implantable cardioverter-defibrillator [38]. However, in the case of a negative PVS, the negative predictive value is at present uncertain, and the whole clinical picture should be considered for therapeutic decisions and advice concerning sports participation [61,62,93].

The European Society of Cardiology (ESC) guidelines on VA and SCD do not provide specific indications on PES for risk stratification in AMVP and primary prevention due to a lack of knowledge in this field [71]. The ESC guidelines on syncope [94] recommend EPS only in patients with unexplained syncope and previous myocardial infarction or other scar-related conditions [95] and suggest it in patients with unexplained syncope preceded by sudden and brief palpitation. These recommendations may be extended to AMVP patients with CMR-proven myocardial fibrosis, especially if the cause of syncope is uncertain and an arrhythmic etiology may be suspected [38].

**Figure 4:**
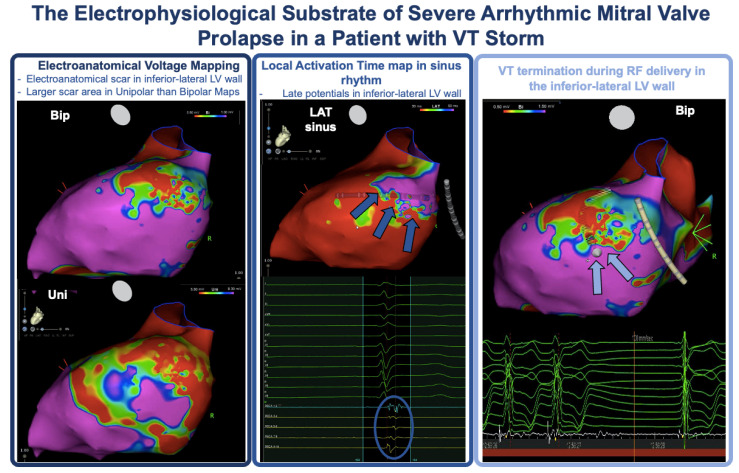
Electroanatomical substrate of a 54-year-old leisure-time sportsman with AMVP presenting with VT storm. There is clear-cut evidence of inferior–lateral perimetral scar with late potentials; radiofrequency energy delivery in the area rapidly led to VT termination.

Recent studies in which electroanatomical voltage mapping was used in patients with AMVP revealed a very high prevalence of abnormal electrograms in the perimitral area (Figure 4).

In the 2016 study by Syed and coworkers, which included AMVP patients with PVC-triggered VF (*n* = 6) or frequent PVCs (*n* = 8), the electroanatomical voltage maps were normal, but in each patient with history of VF or with inducible sustained VT during the EPS, abnormal Purkinje potentials (fractionated, delayed, or mid-diastolic) were recorded, consistent with marked slowing of conduction [52]. In 2021, Vergara and colleagues performed electroanatomical voltage mapping in 15 patients with AMVP and found that a large low unipolar voltage area could be demonstrated in the basal inferolateral LV region in 12 subjects and in the papillary muscle in 5, with concomitant low bipolar voltage in 2 patients. Interestingly, the extension of the abnormal unipolar voltage zone was greater in patients with negative T waves, LGE, and a history of SCD [68]. In a series of 40 patients with AMVP and MAD undergoing catheter ablation of VAs, Ezeddine and colleagues demonstrated a 45% prevalence of abnormal bipolar electrograms (low voltage, fractionated, mid-diastolic, and presystolic potentials), which were most commonly recorded in the anterolateral mitral annulus and/or MAD area (*n* = 10) and the papillary muscles (*n* = 8). Interestingly, in the 10 patients with abnormal bipolar electrograms and available preprocedural CMR, LGE was absent 60% of the time, suggesting that electrogram abnormalities may precede macroscopic fibrosis and that electroanatomical voltage mapping may be a sensitive tool to identify and characterize microscopic fibrosis in the MAD/papillary muscle area [96]. More recently, Chakrabati and colleagues identified low voltage areas in seven out of seven patients with AMVP and MAD undergoing VT ablation and reported that the abnormal voltage area was located in the MAD region in four patients. Interestingly, the low voltage regions were more widespread in unipolar maps compared to bipolar maps. The same authors reported a very high prevalence of low-voltage regions in the posteromedial (94%) and anterolateral (73%) papillary muscles [97].

All these data suggest that electroanatomical mapping may reveal several arrhythmogenic features in patients with a severe AMVP phenotype, similar to what can be found in arrhythmogenic cardiomyopathies [98,99]. However, the clinical role of electroanatomical mapping in the risk stratification process of patients with an intermediate-risk clinical phenotype is at present uncertain, also considering the invasive nature of this test. The finding of low-voltage electrogram areas covering >4 cm^2^ in patients undergoing LV mapping as a preliminary step for VA ablation may be considered a risk feature, especially if late potentials can be recorded, and warrant the discontinuation of competitive sports, as recently suggested in a consensus statement from the Italian society of sports cardiology, although this recommendation has not been validated in the setting of AMVP [84].

## 5. Sports Eligibility Assessment and Therapy

### 5.1. Sports Eligibility

**Figure 5:**
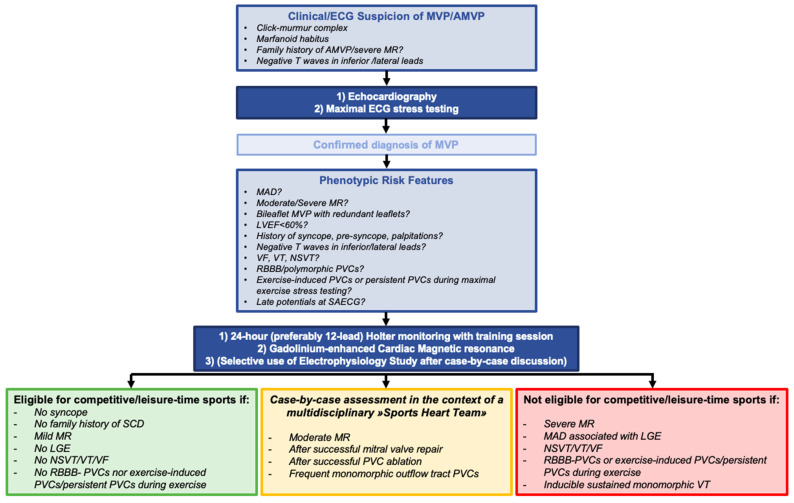
Proposed flowchart for sports eligibility assessment in athletes with MVP.

In most cases, MVP has a benign course (in the absence of moderate-to-severe/severe MR, LV dysfunction/dilation, or an AMVP phenotype), and it does not imply any sports restriction in asymptomatic athletes [100]. Furthermore, a recent retrospective study demonstrated the lack of association between lifetime exercise dose and severe VA or high-risk phenotypic features in MVP patients and that severe Vas most often occur at rest in AMVP patients, therefore suggesting the opportunity to encourage MVP patients to practice moderate intensity exercise, defined by the authors as a total lifetime exercise dose below the threshold of 9.6 MET h/week [30]. However, a comprehensive assessment should be carried out in case of clinical suspicion of MVP, family history of AMVP and/or SCD, symptoms suggestive of Vas, negative T waves/abnormal repolarization at 12-lead ECG, complex/exercise-induced Vas at exercise testing (which is mandatory as part of the preparticipation screening in Italy), as outlined in the preceding sections and recommended by both Cavarretta et al. and Italian guidelines on Sports Cardiology [61,62]. The assessment should include 24 h Holter monitoring with a training session, maximal ECG stress testing, comprehensive TTE, and CMR in case of clinical/imaging clues of an arrhythmogenic phenotype (Figure 5) [61,62].

Competitive athletes with MVP displaying any phenotypic feature of AMVP such as (1) severe MR, (2) MAD associated with basal inferolateral wall fibrosis, (3) personal history of unexplained syncope or family history of SCD at young age, (4) T wave inversion in inferior and/or lateral leads on ECG, and (5) documented complex Vas, should be advised against participating in competitive sports (Figure 5) [60,61]. Athletes with MVP, mild MR, and no phenotypical features of AMVP can participate in competitive or leisure-time sports (class I, level of evidence C); instead, athletes with moderate degenerative MR may participate in competitive or leisure-time sports in selected cases with preserved LV ejection fraction (≥60%), LV end-diastolic diameter <60 mm (or <35.3 mm/m^2^ in men and <40 mm/m^2^ in women), sPAP < 50 mmHg at rest, and a normal maximal exercise stress test (class Iia, level of evidence C) [60]. While Italian guidelines cautiously recommend that patients with severe MR should be advised against participating in competitive sports (class III), European guidelines suggest that engagement in leisure-time or competitive sports may be considered (class Iib, level of evidence C) in the same circumstances as patients with moderate MR (preserved LV ejection fraction, LV end-diastolic diameter <60 mm, sPAP < 50 mmHg at rest, and a normal maximal exercise stress test) [60]. In the end, borderline cases should be collegially evaluated at referral centers, using a “sports heart team” approach and considering patients’ values in a shared decision-making process (Figure 5) [60,84].

### 5.2. Pharmacological Treatment

Antiarrhythmic drug treatments may be effective in controlling symptoms and improving LV function in AMVP patients with frequent PVCs. In a recent series of patients with AMVP in which Vas were refractory to beta-blocker treatment, which is a common first-line option for a wide range of arrhythmias [101], the combination of flecainide and low-dose beta-blocker resulted in a marked reduction in PVC and NSVT burden [102]. However, antiarrhythmic drugs are usually not tolerated or undesired by athletes, due to the profound resting sinus bradycardia seen in (especially endurance) athletes, the risk of impaired exercise performance, and therefore lack of therapeutic tolerance. Catheter ablation may represent an important treatment option for athletes with AMVP [33,60].

### 5.3. PVC/VT Ablation

Although challenging, ablation of foci within the papillary muscles has been reported to be effective, and long-term success rates for PVC ablation in MVP range between 60 and 84% [52,53,68,69,96,97,103,104,105]. The development of catheters with contact force sensors, the use of intracardiac echocardiography, which improves catheter positioning on the PM, or cryoablation catheters that ensure better catheter stability during freezing, has led to improved success rates [103,104,105,106], and the continuous ideation and clinical validation of novel catheter platforms (i.e., catheters allowing ablation with different energy sources [such as electroporation], higher radiofrequency energy settings, improved irrigation) may further ameliorate procedural outcomes in future years [107,108].

In both the recent studies by Ezzeddine et al. and by Chakrabati et al., the efficacy of CA in reducing PVC burden and the risk of recurrent sustained VAs in AMVP with MAD was confirmed with mid–long-term follow-ups [96,97]. Ezzeddine and colleagues also reported that substrate modification in the region of MAD was associated with a trend toward reduced probability of reablation during follow-up, suggesting that substrate ablation might be helpful in the presence of pathological slow conduction in the region of MAD [96].

According to both current ESC guidelines for the management of patients with VAs and the prevention of SCD and the EHRA AMVP expert consensus statement, ablation is recommended in the following situations [5,71,109]:

(1) Ablation of PVCs that trigger recurrent VF;
(2) Ablation of sustained monomorphic VT in cases of recurrent ICD therapies despite antiarrhythmic drug therapy or when pharmacological therapy is contraindicated or not desired/tolerated (Figure 4);
(3) PVC ablation in symptomatic patients, either as an alternative to antiarrhythmic drug treatment or when drugs are not desired, not tolerated, or ineffective;
(4) PVC ablation in patients with PVC-induced LV dysfunction.

Although PVC is often implicated in VF initiation in AMVP, the concept that PVC ablation may reduce the risk of SCD in patients without a prior aborted SCD episode remains unproven. Furthermore, there is currently a paucity of data concerning the reassessment of sports eligibility after catheter ablation for PVCs or hemodynamically tolerated VTs in the setting of AMVP. However, in case of outflow tract or fascicular VAs, in the absence of other phenotypic risk features, when the ablation is effective, and the athlete is rendered asymptomatic, competitive sports may be restarted after comprehensive reassessment, including repeated 24 h Holter monitoring and maximal exercise stress testing showing absence of complex/exercise-induced VAs [60]. In the case of left-sided ablations, in which an oral anticoagulant is usually prescribed for 4 weeks, contact sports should be restarted after interruption of the anticoagulant to minimize the risk of bleeding.

### 5.4. ICD Implantation

The general principles concerning ICD implantation also apply to patients with AMVP [65]. The secondary prevention of ICD is indicated by guidelines in patients with AMVP and a documented history of SCD with VF or VT without other clear reversible causes [71].

Following the EHRA expert consensus statement on AMVP and MAD complex, an ICD should be considered for MVP patients with unexplained syncope and sustained VT likely arising from the mitral apparatus [5]. The option of an ICD may be reasonable for AMVP patients with unexplained syncope and NSVT (documented by ECG, Holter monitoring, ILR, or exercise testing), but also in AMVP patients with 1 high-risk feature (hemodynamically tolerated sustained VT, NSVT, or unexplained syncope) and two or more phenotypic risk features (T-wave inversion in inferior leads, repetitive polymorphic PVCs, MAD, redundant MV leaflets, enlarged LA or an LVEF ≤ 50%, and LGE within the mitral apparatus) [5]. In young subjects, a subcutaneous ICD may be preferable to avoid long-term complications of transvenous leads [110]. Participation in competitive sports should be discouraged in AMVP ICD carriers according to both European and Italian guidelines [60,61].

### 5.5. Surgical Correction

Although early MV repair is key for restoring a normal life expectancy in patients with MVP and severe MR [17], the impact of surgery on the risk of life-threatening arrhythmias in AMVP patients remains controversial. Several case reports have reported a reduced burden of Vas [111], but the long-term outcomes and the impact of different surgical techniques remain uncertain [112,113]. Theoretically, MV repair may eliminate MAD, and interrupt the maladaptive mechanical process underpinning papillary muscle/inferolateral fibrosis [5,62]; furthermore, the cryoablation of VAs may be performed during surgical MV repair [5]. However, a recent case series reported that although MV surgery resulted in a reduction in VA burden in 55% of AMVP patients, 19% of patients without VAs prior to MV repair developed complex VAs during follow-up, warranting continued surveillance [114]. Return to play after successful MV repair should be evaluated on a case-by-case basis, in the absence of residual moderate/severe MR, LV dysfunction, and arrhythmias at 24 h Holter monitoring and maximal exercise stress testing [61,62]. Contact sports should be cautiously restarted after the initial three months of anticoagulant treatment with vitamin K antagonist in patients without long-term indications for anticoagulation.

## 6. Gaps in Knowledge and Future Directions

Despite the considerable amount of work carried out in the field of AMVP during recent years, several issues still need to be carefully assessed. In particular, the safe prescription of physical exercise in AMVP patients is still uncertain. Recommended levels of exercise to maximize the health benefits derived from physical activity have been reported at 7.5 MET h/week, and the study by Five et al. suggested that the risk of severe VAs does not increase until 9.6 MET h/week [30], but dedicated prospective studies involving MVP patients are still needed to confirm these recommendations. The prognosis of PVC morphologies traditionally considered to be common and indicate low risk of SCD among athletes (i.e., monomorphic outflow tract/fascicular PVCs) remains to be assessed in the setting of AMVP [5,33], especially considering that a specific association of PVCs morphologies compatible with origin from right ventricular outflow tract alternating with fascicular system/papillary muscle PVCs was found to be common in AMVP patients with prior aborted SCD [66]. Although genetic testing may be considered for AMVP with familiar clustering or syndromic stigmata, further studies should be conducted on the topic [42]. Furthermore, the proposed criteria for ICD implantation in primary prevention need prospective validation [5]. The prognostic roles of EPS and EAM, both in the general population and for sports eligibility assessment, are still to be ascertained in dedicated studies. Finally, the prognosis and sports eligibility assessment of MAD in the absence of MVP, which accounted for 22% of all MAD identified in a large observational study [83], remains unclear. Future studies should try to combine phenotypic risk features into a clinical tool (i.e., a risk score), which could simplify patient management and help in the selection of ICD candidates [71].

## 7. Conclusions

MVP is a common condition and generally has a benign course, but given the possible association between MVP and SCD, it is mandatory for identifying the subgroup of patients at risk of malignant VAs. Preparticipation screening with history, physical examination, 12-lead ECG, and possibly exercise stress testing may be of paramount importance to intercept AMVP athletes with phenotypic features of high risk who require further evaluation and appropriate management [60,61]. Further longitudinal studies are needed to inform sports prescription and to upfront identify the highest-risk patients who could potentially benefit from a primary prevention ICD implantation.

## Figures and Tables

**Table 1:** Summary of the main studies reporting the prevalence of MVP in autopsy series of SCD in athletes. SCD: sudden cardiac death; MVP: mitral valve prolapse.

Study	Year	Population	Total No. SCD	Sports-Related SCD	Age	Male Sex	Total No. of MVP	Total No. of MVP in Athletes
Burke et al. [25]	1991	Study of sports and non-sports-related SCD	690	34 (5%)	14–40 years	532 (77%)	11 (1.6%)	0 (0%)
Van Camp et al. [26]	1995	Study of sports-related death in athletes	100	100	13–22 years	92 (92%)	1 (1%)	1 (1%)
Maron et al. [27]	1996	Registry of SCD in young athletes	134	134	<35 years	120 (89.5%)	3 (2.24%)	3 (2.24%)
Maron et al. [28]	2009	Registry of SCD in young athletes	1049	1049	13–25 years	937 (89.3%)	25 (2.4%)	25 (2.4%)
Maron et al. [29]	2014	Registry of SCD in young athletes	47	47	18–22 years	41 (87%)	1 (2.1%)	1 (2.1%)
Finocchiaro et al. [23]	2016	Registry of SCD in athletes	357	357	7–67 years	330 (92%)	7 (2%)	7 (2%)
Maron et al. [24]	2016	Registry of SCD in young athletes	842	842	13–25 years	747 (89%)	31 (4%)	31 (4%)

**Table 2:** The main clinical studies on clinical features of AMVP in athletic populations.

Study	Year	Population	No. of Patients	MVP	Age	Male Sex	VAs in MVP	SCD/Aborted SCD	ICD Shock	Sustained VT
Caselli et al. [22]	2018	Athletes	7449	215 (2.9%)	17–43 y	67%	62 (29%)	0 (%)	0 (0%)	0 (0%)
Five et al. [30]	2023	Athletes	136	136 (100%)	37–64 y	49%	17 (12.5%) [severe VAs]	12 (8.8%)	1 (0.74%)	4 (2.9%)

## Data Availability

No new data were created or analyzed in this study. Data sharing is not applicable to this article.
